# Impact of red-to-blue LED light ratios on the yield and phytochemistry of leafy green amaranths

**DOI:** 10.3389/fpls.2026.1957692

**Published:** 2026-10-30

**Authors:** Tori Rosen, A.J Both, Qingli Wu, James E. Simon

**Affiliations:** 1 New Use Agriculture and Natural Plant Products, Department of Plant Biology, Rutgers University, New Brunswick, NJ, United States; 2 Department of Environmental Sciences, Rutgers University, New Brunswick, NJ, United States

**Keywords:** *Amaranthus tricolor*, baby greens, CEA, climate resilient crops, hydroponics, leafy greens, nutrient density, secondary metabolites

## Abstract

Leafy green amaranth (*Amaranthus tricolor)* is a crop of global importance due to its vigorous growth in resource-limited environments and rich nutritional content. Increased human migration to urban centers across the United States brings demands for culturally appropriate food items that meet the culinary preferences of incoming international communities. Leafy green amaranth is commonly eaten across South and East Asia, Sub-Saharan Africa, Latin America, and the Caribbean, and is therefore an important candidate for marketing to diverse urban populations interested in culturally relevant produce. Our study assessed the impact of different light spectra delivered to three accessions of baby amaranth greens grown in a hydroponic system installed in a growth chamber. The delivered light spectra had red:blue (R:B) ratios including 1:1.6, 1:1.1, 1.5:1, and 1.9:1. Plant yield traits assessed included shoot height, shoot fresh mass, number of leaves, and leaf area of 6-week-old baby greens, as well as nutritional traits including carotenoids, chlorophylls, betalain pigments, phenolic acids, and flavonoids. Our study found small differences in yield caused by the different R:B ratios, but significant differences in nutritional content. Results can be used to maximize the nutritional content of amaranth, without compromising leafy green yield.

## Introduction

Increased immigration into the United States, particularly in urban areas, has created opportunities for globally important crops not previously widely available for sale at specialty ethnic urban markets (Joassart-Marcelli et al., 2017; Govindasamy et al., 2022). The increased market demand in turn creates opportunities for production of these specialty crops within urban environments, whether that be home and community gardens or indoor hydroponic systems (Diekmann et al., 2020; Oh and Lu, 2023). Leafy green amaranth (*Amaranthus* spp.) is a culturally important crop among various immigrant communities in the United States (Govindasamy et al., 2022). It is of global importance, particularly throughout Sub-Saharan Africa, Mesoamerica, South and East Asia, and the Caribbean due to its historical use as a nutrient-dense, climate resilient crop (Das, 2016). As such, amaranth greens have great potential to be integrated into urban agriculture and food systems (Rosen et al., 2024).

Leafy green amaranth is renowned for its high nutritional density, particularly relating to primary and secondary metabolites including betalain pigments, phenolic acids, flavonoids, carotenoids, and chlorophylls (Venskutonis and Kraujalis, 2013). The betalain pigments, red/violet betacyanins and yellow/orange betaxanthins, are unique to the *Caryophyllales* order and have similar antioxidant properties to anthocyanins but are stable at a higher pH range (Sadowska-Bartosz and Bartosz, 2021). Betalains, particularly betanin, are well-studied in beet root, but amaranth is emerging as an alternate source because of its unique amaranthin and isoamaranthin betacyanin pigments (Howard et al., 2022). Amaranths also contain hydroxycinnamic acid-derived antioxidant compounds, particularly caffeic acids, ferulic acids, coumaric acids, and flavonoids, such as quercetin and kaempferol derivatives (Schröter et al., 2018). Hydroxycinnamic acids and flavonoids have been associated with lower occurrences of cardiovascular and noncommunicable diseases such as diabetes, cancers, and age-related neurodegenerative conditions (Kumar and Pandey, 2013; Coman and Vodnar, 2020). Carotenoids and chlorophylls are photosynthetic pigments with antioxidant properties, particularly carotenoids which are found in high amounts in amaranths (Pérez-Gálvez et al., 2020; Nazeer et al., 2022). Each of these compounds are synthesized in amaranth through biochemical processes and are impacted by genetic factors and environmental conditions (Saini and Keum, 2018).

Baby leaf vegetables are young, tender vegetables harvested before their mature vegetative stage. Baby greens are gaining attention due to their rapid harvest cycle, nutrient-density and minimal post-harvest processing required for fresh consumption (Saini et al., 2017). Indoor farms require quick, energy-efficient production strategies from seed to harvest to ensure profitability, while maintaining overall marketability through product quality. Researching environmental effects on baby leaf vegetables has also been used as a means to predict the overall performance of mature greens (Lee et al., 2026). The short-term effects that sole-source light spectral manipulation has on immature greens can either determine marketability of these baby leafy vegetables or be used as an indicator of overall plant performance at a mature stage. Therefore, it is important to understand and optimize plant growth and development during an immature stage in energy-intensive indoor production environments.

In indoor agriculture with sole-source lighting, spectral manipulations are often explored as a means to increase bioaccumulation of secondary metabolites (Thoma et al., 2020). Primary and accessory photosynthetic pigments including chlorophylls and carotenoids absorb red to blue light within the photosynthetically active radiation (PAR) range (400–700 nm), resulting in plant growth and development. Secondary metabolites such as flavonoids, phenolic acids, and betalain pigments are synthesized downstream of the shikimate pathway and have a suggested increased biosynthesis under UV radiation (280-400) and light stress (Saito et al., 2013; Liu et al., 2023).

Our paper aims to elucidate the effect that variations in red to blue (R:B) light ratios has on yield and the accumulation of primary and secondary metabolites of three accessions of *Amaranthus tricolor* baby greens in a hydroponic system. While previous studies have examined the effects of monochromatic red and blue lights when compared to combinations of the two, we aim to evaluate whether moderate differences in R:B ratios will still show a significant effect in the growth and phytochemistry of *Amaranthus tricolor* accessions. We hypothesize that moderate differences in the R:B ratio will create physiological and metabolic differences among the three accessions, and that these effects will vary significantly among the three accessions.

## Materials and methods

### Plant material and growing conditions

This study was conducted in an EGC GC-15 reach-in growth chamber (New Brunswick, NJ; 40°28’42” N, 74°26’15” W) from January to October, 2025. Three *Amaranthus tricolor* accessions were evaluated in a completely randomized design, with the study replicated three times. Light fixtures remained constant through the three repeated studies, but accessions were randomly positioned under the lights for each of the three grow cycles. Three *A. tricolor* Rutgers breeding lines were evaluated in this study: A white-stemmed accession (Am90), a bicolored accession (Am25), and a red accession (Am98). Seeds were sown on January 20^th^, June 12^th^, and August 15^th^ 2025 in a peat moss vermiculite substrate (1:1 mixture) in two full-size 24-cell seedling trays and two 24-cell trays cut in half to total 72 cells, which were placed directly into the growth chamber. Germination occurred within 7 days after sowing and the seedlings were thinned 12 days after germination to ensure only one plant per cell remained. A total of 72 plants (24 subsamples within each accession) were grown within each replicated trial. Plants were bottom irrigated once daily at 9:00 AM using the ebb and flow technique, powered by a 115V AC Little Giant Centrifugal Pump (Franklin Electric; Fort Wayne, IA, USA). Peter’s Excel 21N-5P-20K water soluble fertilizer (Scotts Company; Marysville, OH, USA) was used with every irrigation, adjusting the pH to 5.5-6.5 with Na_2_CO_3_ and maintaining an electrical conductivity (EC) of 1.2-1.7 mS cm^-1^. The growth chamber temperature set points ranged from 19 °C to 23 °C and an average relative humidity of 64 ± 5.4%.

### Sole-source lighting

The growth chamber was outfitted with four California Lightworks Solarsystem 550 LED fixtures (Canoga Park, CA), each with adjustable red, white, and blue light intensities. Plants received sole-source light for 16-hours each day throughout the duration of the studies. Germination took place under a daily light integral (DLI) of 13.6 ± 0.7 mol m^-2^ d^-1^ with a uniform light spectrum within the growth chamber (**Supplementary Figure 1A**). The DLI for the second week was 17.0 ± 0.9 mol m^-2^ d^-1^, again with a uniform light spectrum (**Supplementary Figure 1B**). From week 3 to week 6, the three accessions were treated with a gradient light spectrum by adjusting the output of each fixture separately. Treatment 1 had a R:B ratio of 1:1.6, Treatment 2 had a R:B ratio of 1:1.1, Treatment 3 had a R:B ratio of 1.5:1, and Treatment 4 had a R:B ratio of 1.9:1 (**Table 1**; **Figure 1**). The PAR levels were measured using a PAR meter (Model MQ-500, Apogee Instruments; Logan, UT), the spectral data were collected using a Gigahertz-Optik spectroradiometer (Model BTS256-EF, Türkenfeld, Germany), and the spectral settings of the fixtures were entered into the controllers for consistency across the three replicated studies. The average DLI for weeks 3–6 was 20.2 ± 1.2 mol m^-2^ d^-1^ across all four treatments.

**Table 1:** Spectral distribution of the average PAR and DLI measured directly below each of the four LED fixtures at the top of the plant canopy.

Treatment	Blue (400–500 nm) (W m^-2^ nm^-1^)	Green (500–620 nm) (W m^-2^ nm^-1^)	Red (620–700 nm) (W m^-2^ nm^-1^)	R:B ratio	Total DLI (mol m^-2^ d^-1^)
1	29.7 ± 1.1	27.2 ± 1.8	19.0 ± 1.1	1:1.6	19.9 ± 1.1
2	24.4 ± 1.2	27.0 ± 1.8	21.8 ± 1.1	1:1.1	19.6 ± 1.1
3	18.7 ± 1.2	25.6 ± 1.9	28.9 ± 1.8	1.5:1	20.2 ± 1.3
4	17.6 ± 1.2	24.8 ± 1.8	33.6 ± 1.4	1.9:1	21.3 ± 1.2

The plants were exposed to a 16-hour photoperiod.

**Figure 1:**
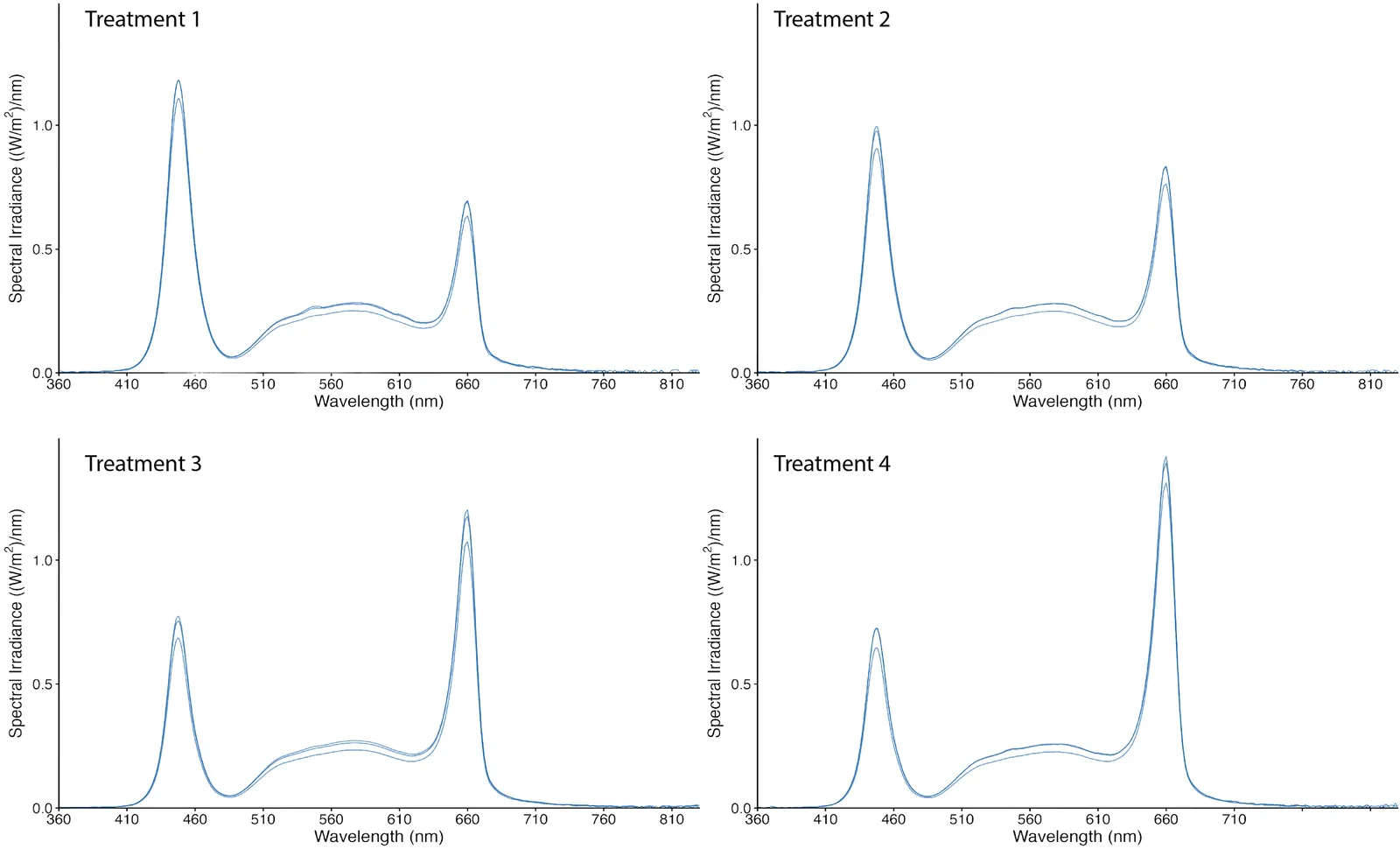
Average (n = 12) light spectra measured directly below each of the four LED fixtures at the top of the plant canopy. Trt1 = 1:1.6 R:B, Trt2 = 1:1.1 R:B, Trt3 = 1.5:1 R:B, Trt4 = 1.9:1 R:B.

### Plant morphological data collection

Plants were harvested 38 days after sowing, on February 27, July 20, and September 21, 2025, respectively. All 24 plants were evaluated as subsamples for each accession. Morphological traits evaluated were shoot height (cm), number of mature leaves, shoot fresh mass (g), and leaf area (cm^2^). Mature leaves were identified as leaves that were unfurled. Five random leaves from each accession within each treatment group were evaluated for leaf area, which was estimated using ImageJ software (Schneider et al., 2012). The remaining plant tissue from each accession was aggregated and kept in a -80 °C freezer to be prepared for phytochemical analysis.

### Sample preparation for phytochemical analysis

The aggregated frozen plant tissue for each accession under each treatment was lyophilized and ground into a fine powder for extraction. The three replicated trials were analyzed separately. Approximately 50 mg of dried ground plant material was extracted with 5 mL 70% MeOH + 0.1% formic acid in water for phenolic acids and flavonoid quantification, 5 mL 100% water extraction for betacyanin and betaxanthin pigment quantification, and 5 mL 100% MeOH extraction for carotenoid and chlorophyll quantification. Plant extracts were kept in a -20 °C freezer until analysis. Plant extracts were further diluted to a final concentration of 2 mg mL^-1^ for LC-UV-MS analysis.

### Total carotenoids and chlorophylls

Total carotenoids and chlorophyll A and B were quantified in triplicate following the methods of Lichtenthaler and Buschmann (2001), using their mean molar coefficient equations for 100% methanolic extracts. Total chlorophylls were quantified by adding together chlorophyll A and chlorophyll B values. UV absorbance values were measured at 665.2 nm, 652.4 nm, and 470 nm using a Biotek Epoch Microplate Spectrophotometer (Agilent; Santa Clara, CA). Values were recorded as µg g^-1^ dry mass.

### Total betacyanin and betaxanthin content

Total betacyanin and betaxanthin pigments were quantified in triplicate following the methods of Wyler et al. (1959) using dried plant material extracted in 100% water. Betacyanin pigments were measured at 540 nm and betaxanthins at 470 nm using a Biotek Epoch Microplate Spectrophotometer (Agilent; Santa Clara, CA). The mean molar extinction coefficients of 62•10^6^ cm^2^ mol^-1^ and 48•10^6^ cm^2^ mol^-1^ were used to calculate total betacyanin and betaxanthin content, respectively, using Beer's Law. Values were recorded as ng g^-1^ dry mass.

### Total phenolics

Total phenolics were quantified in triplicate using the Folin-Ciocalteau’s total phenolics (FC) assay, modified from Singleton and Rossi (1965). Gallic acid was serially diluted 2-fold, starting at 100 mg mL^-1^and diluted 64 times to reach a final concentration of 1.56 mg mL^-1^. Spectrophotometric absorbance was measured at 762 nm using a Biotek Epoch Microplate Spectrophotometer (Agilent; Santa Clara, CA). Values were reported as mg g^-1^ dry mass.

### UHPLC-DAD-QTOF/MS compound identification

The separation and identification of betacyanin pigments, phenolic acids, and flavonoids was performed using an Agilent 1290 Infinity II UHPLC system (Santa Clara, CA) coupled with a diode array detector (DAD) and quadruple time-of-flight (QTOF) mass detector. The electrospray ion mass spectrometer was under negative mode in separated runs with a Phenomenex Luna C18(2) 150 x 4.60 mm, 5µm column (Torrance, CA). The column temperature was at 25 °C. The mobile phase consisted of 100% water with 0.1% formic acid (Eluent A) and 100% acetonitrile with 0.1% formic acid (Eluent B). The gradient elution was as follows: 0–3 min 5-10% B; 3–10 min 10% B; 10–30 min 10-32% B. The flow rate was set to 0.5 mL min^-1^, and the injection volume was 4.0 µL. The MS parameters were as follows: Gas temperature of 320°C; gas flow of 8 L min^-1^; nebulizer pressure of 241.3 kPa; sheath gas temperature of 200°C; sheath gas flow of 11 L min^-1^; capillary voltage of 3,500 V; skimmer voltage of 65 V; collision energy of 30 V; and fragmentor voltage of 175 V.

### LC quantification

The quantification of phenolic acids and flavonoids used the same Agilent 1290 Infinity II UHPLC-DAD-QTOF/MS system, with the same column and mobile phase parameters that were used for compound separation and identification. Samples were quantified in duplicates. Derivatives of caffeic acid, ferulic acid, and coumaric acid were quantified at 320 nm; quercetin and kaempferol derivatives were quantified at 370 nm. The phenolic and flavonoid compounds were semi-quantified by using a chlorogenic acid (0.13-16.67 µg mL^-1^; R^2^ > 0.99), ferulic acid (0.14-17.67 µg mL^-1^; R^2^ > 0.99), and quercetin-3-rutinoside (0.13-16.67 µg mL^-1^; R^2^ > 0.99) standard curve. Values were reported as µg g^-1^ dry mass.

### Statistical analysis

To test for significant differences across treatments, a blocked two-way factorial ANOVA analysis examining treatment x accession interactions was performed on the raw values within the three repeated experiments for all yield and nutritional traits, with a threshold of P < 0.05 for significant difference. For traits that differed significantly across light treatments, Fisher’s LSD test from R package emmeans (estimated marginal means) was performed to determine which treatments were significantly different from one-another.

A second model testing the effect of Red: Blue ratio, Red:(Green+Blue) ratio, and daily light integral (DLI) was performed to identify the source of variation within each treatment. P values that were significant from the original treatment x accession blocked two-way factorial ANOVA (Model A) were compared to significant P values from isolated light spectral variables (Model B) to determine whether significant differences were truly due to the R:B ratio or to other parameters within the experimental design.

## Results

### Plant growth and yield

Plant yield traits were measured 38 days after sowing, including shoot height (cm), number of mature leaves, shoot fresh mass (g), and leaf area (cm^2^). Of these traits across the three accessions evaluated, only shoot height showed significant differences from the treatment effect within Am25, with a range of 6.61-8.0 cm, although the spectral and DLI effects were not significant (**Figure 2A**; **Supplementary Table 1**). Across Am25 and Am90, Treatment 3 resulted in the shortest plants averaging 6.61 ± 0.6 cm for Am25, and 3.71 ± 0.3 cm for Am90. Treatment 4 produced the shortest plants for Am98 (7.71 ± 0.6 cm). Similar trends were observed for shoot fresh mass measurements (**Figure 2B**), with Treatment 3 resulting in the lowest fresh mass for Am90 (1.06 ± 0.2 g) and Treatment 4 having the smallest fresh mass for Am98 (1.15 ± 0.2 g). Am25, however, had a lower fresh mass for Treatment 4 compared to Treatment 3, although not significantly lower. The number of mature leaves varied by one leaf in Treatment 2 for Am25, and again in Treatments 2 and 4 in Am90, but these differences were not significant (**Figure 2C**). There were no detectable trends in leaf area under the different treatments, though differences across accessions were significant (**Figure 2D**; **Supplementary Table 1**). The highest average leaf area for Am25 was under Treatment 1 (15.3 ± 3.1 cm^2^) and lowest was under Treatment 4 (9.5 ± 1.0 cm^2^). For Am90, the highest average leaf area was under Treatment 4 (11.7 ± 2.2 cm^2^) and the lowest average under Treatment 3 (7.2 ± 2.5 cm^2^). For Am98, the highest average leaf area was under Treatment 3 (13.0 ± 1.0 cm^2^) and the lowest average was under Treatment 2 (10.0 ± 1.1 cm^2^). Light spectrum and DLI as isolated variables had a significant effect on leaf area for Am98 at P < 0.05 (**Supplementary Table 1**). There were significant genotypic differences between the three accessions, with shoot height, number of leaves, and leaf area varying significantly between the accessions (**Supplementary Table 1**), however, the delivered R:B light ratios did not significantly affect the yield of the plants.

**Figure 2:**
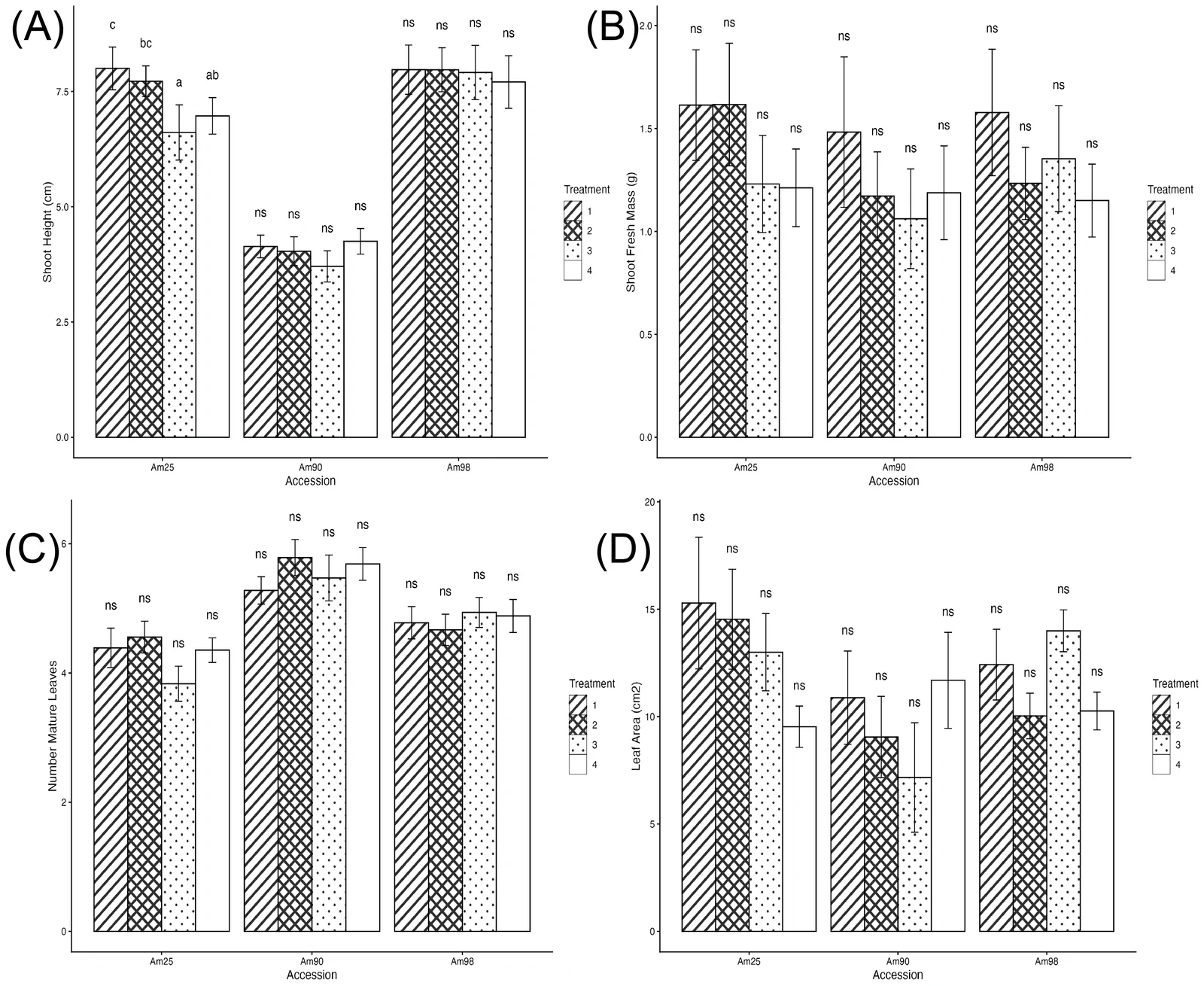
Average yield measurements (n=72) for three accessions of *Amaranthus tricolor* grown under different R:B ratio treatments. **(A)** shoot height (cm); **(B)** shoot fresh mass (g); **(C)** number of mature leaves; **(D)** leaf area (cm^2^). Letters indicate significantly different values at P < 0.05 as evaluated by Fisher’s LSD test. Traits that do not differ significantly at P < 0.05 are labeled ns.

### Total carotenoids and chlorophylls

Across the four R:B treatments delivered to the three amaranth accessions, total carotenoids, chlorophyll A, and total chlorophyll varied significantly in overall treatment effects, while chlorophyll B varied significantly for Am25 and Am90, but not for Am98. All three accessions had significantly higher carotenoid levels under Treatment 3, but the light spectrum or DLI effects were only significant for Am90 and Am98 (**Figure 3A**; **Supplementary Table 1**). On average, total carotenoids ranged from 33.35 – 59.33 µg g^-1^ dry mass across all accessions, with the lowest carotenoid levels varying by treatment across the three accessions. The lowest level of carotenoids for Am25 occurred under Treatment 1 (38.92 ± 3.37 µg g^-1^ dry mass), under Treatment 2 for Am90 (33.75 ± 0.93 µg g^-1^ dry mass), and under Treatment 4 for Am98 (33.35 ± 3.25 µg g^-1^ dry mass). Similar trends were observed for chlorophyll A and B and total chlorophylls. Across all accessions, chlorophyll A ranged from 121.41 – 210.18 µg g^-1^ dry mass (**Figure 3B**) and chlorophyll B ranged from 39.20 – 56.49 µg g^-1^ dry mass (**Figure 3C**). Isolated spectral or DLI effects were significant in Am90 and Am98, but not Am25 for chlorophyll A, and were only significant in Am90 for chlorophyll B (**Supplementary Table 1**). Similar to total carotenoids, Am25 accumulated the lowest chlorophyll levels under Treatment 1, Am90 had the lowest levels under Treatment 2, and Am98 had the lowest chlorophyll levels under Treatment 4 (**Figure 3D**). All three accessions had significantly higher total chlorophyll levels under Treatment 3, with total chlorophyll values ranging from 225.05 – 266.66 µg g^-1^ dry mass compared to a range of 170.73 – 226.11 µg g^-1^ dry mass under Treatments 1, 2, and 4. Differences in total chlorophyll were significantly affected by light spectrum or DLI for Am90 and Am98, but not Am25 (**Supplementary Table 1**).

**Figure 3:**
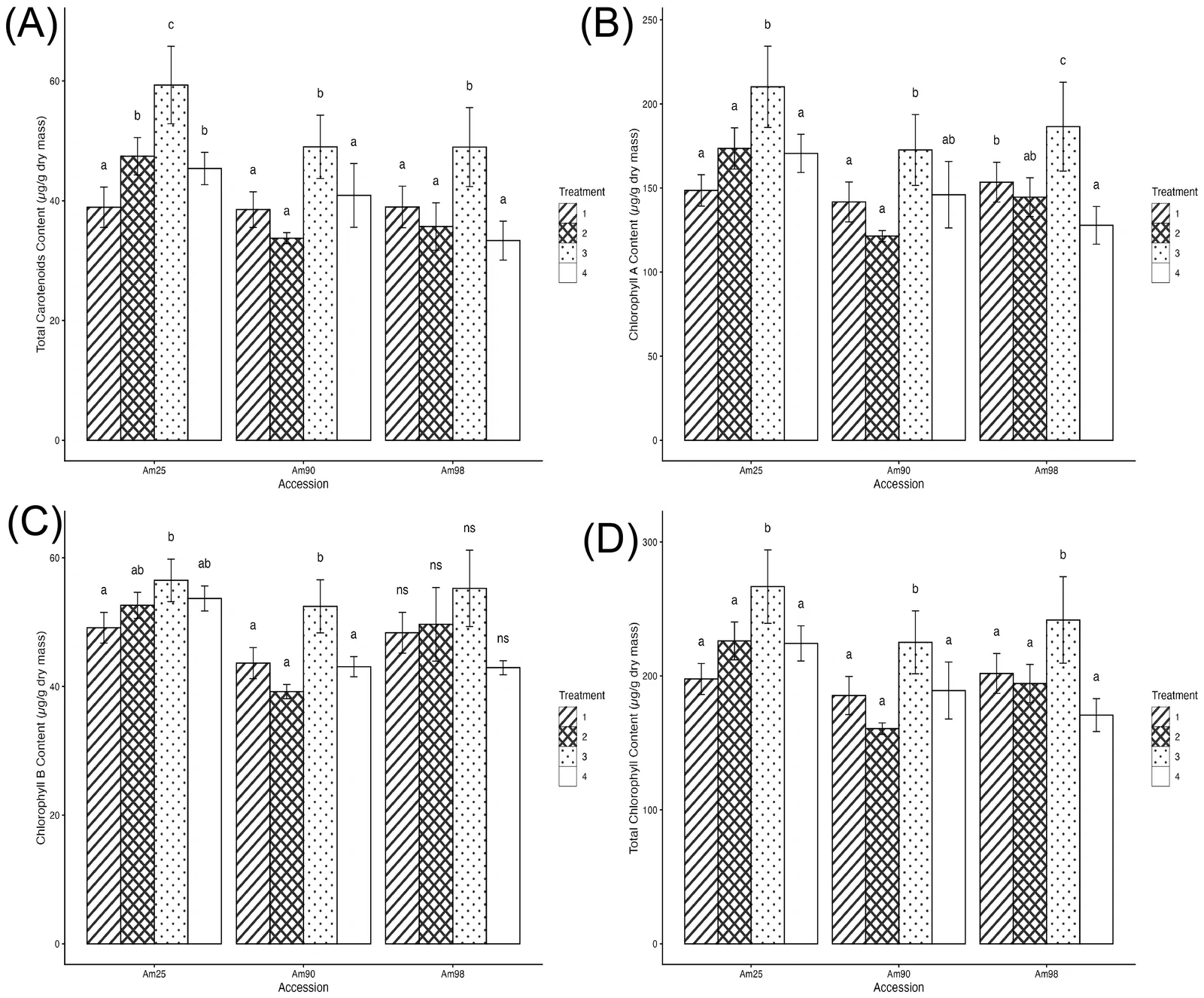
Average (n=12) photosynthetic pigment content of three accessions of *Amaranthus tricolor* grown under different R:B ratio treatments. **(A)** total carotenoids content (µg g^-1^ dry mass); **(B)** chlorophyll a content (µg g^-1^ dry mass); **(C)** chlorophyll b content (µg g^-1^ dry mass); **(D)** total chlorophyll content (µg g^-1^ dry mass). Letters indicate significantly different values at P < 0.05 as evaluated by Fisher’s LSD test. Traits that do not differ significantly at P < 0.05 are labeled ns.

### Betalain pigments, phenolic acids, and flavonoids

The three accessions varied significantly in betacyanin and betaxanthin pigment accumulation. Betacyanin and betaxanthin pigment content varied significantly across treatments only in the fully red accession, Am98, ranging from 198.03 – 267.78 and 130.40 – 175.53 ng g^-1^ dry mass, respectively (**Figures 4A, B**). Am25, a bicolored amaranth with visible red pigment on the leaves and stems, had its measured betacyanin content range from 63.56 – 70.62 ng g^-1^ dry mass and its betaxanthin content range from 77.47 – 89.70 ng g^-1^ dry mass, although these values did not differ significantly across treatments. Am90, a white-stemmed, green leafed accession with no visible red pigmentation, had a measured betacyanin content ranging from 22.21 – 29.41 ng g^-1^ dry mass, even though this accession’s betacyanin content did not vary significantly. Total betaxanthin content in Am90 was measurably higher, ranging from 48.90 – 57.76 ng g^-1^ dry mass. In accessions Am25 and Am98 with visible red pigmentation, the lowest recorded betacyanin content was observed under Treatment 4, although it was only significantly lower in Am98 compared to treatments 1 and 3. The highest betacyanin content of Am25 and Am98, the two accessions with visible red pigmentation, was observed under Treatment 3, although it was not significantly higher than the betacyanin accumulation under Treatment 1. The betaxanthin content was the lowest under Treatment 4 and highest under Treatments 1 and 2, though only significantly higher for Am98 (**Figure 4B**).

**Figure 4:**
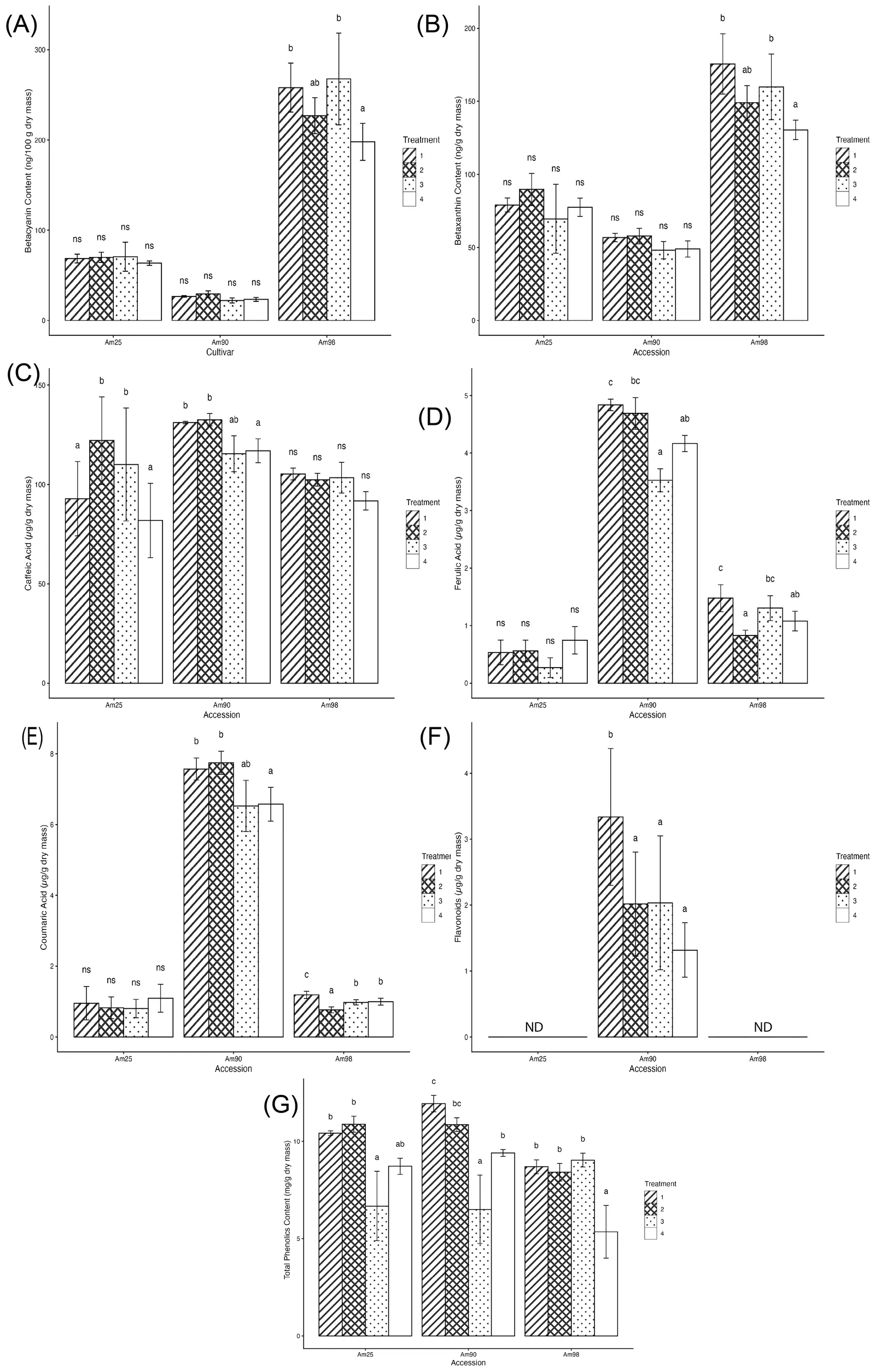
Average (n=12) secondary metabolite content of three accessions of *Amaranthus tricolor* grown under different R:B ratio treatments. **(A)** total betacyanin content (ng g^-1^ dry mass); **(B)** total betaxanthin content (ng g^-1^ dry mass); **(C)** total caffeic acid content (µg g^-1^ dry mass); **(D)** total ferulic acid content (µg g^-1^ dry mass); **(E)** total coumaric acid content (µg g^-1^ dry mass); **(F)** total flavonoid content (µg g^-1^ dry mass). ND indicates amounts too low to detect; **(G)** total phenolics content (mg g^-1^ dry mass). Letters indicate significantly different values at P < 0.05 as evaluated by Fisher’s LSD test. Traits that do not differ significantly at P < 0.05 are labeled ns.

Phenolic acids and flavonoids found in the leafy green amaranth accessions were identified as derivatives of caffeic acid, ferulic acid, and coumaric acid (**Supplementary Figure 2**) and flavonoids identified included quercetin-3-O-rutinoside, quercetin-3-O-glucoside, and kaempferol-3-O-rutinoside (rutin, isoquercetin, and nicotiflorin, respectively) based on the UV absorption and high resolution mass spectrometry (HRMS). Caffeic acids were the most abundant phenolic acids across all three accessions, ranging from 81.85 – 132.46 µg g^-1^ dry mass (**Figure 4**). Caffeic acid levels were significantly higher under Treatments 1 and 2 with Am90 and Treatment 2 and 3 with Am25, but there were no significant differences observed with Am98 (**Figure 4C**). Ferulic and coumaric acids were found in much lower quantities than caffeic acid, ranging from 0.27 – 4.84 and 0.77 – 7.75 µg g^-1^ dry mass, respectively. Significant differences were observed in Am90 and Am98 across treatments for ferulic and coumaric acids, with generally higher bioaccumulation of these phenolic acids under Treatments 1 and 2 (**Figures 4D, E**). The highest values of ferulic acid were observed under Treatment 1 for Am90 and Am98. For Am25, however, the highest ferulic acid content was observed under Treatment 4, although not significantly higher. Coumaric acids were most abundant under Treatment 2 of Am90 and Treatment 1 for Am98 and were significantly higher than under Treatments 3 and 4. Again, the differences were not significant for Am25, but there was an increase in coumaric acid content under Treatment 4. Ferulic acid content had a significant Treatment x Accession interaction, and DLI was the only parameter with a significant effect on ferulic acid for Am90, while the light spectral ratios and DLI had a significant effect on ferulic acid in Am98 (**Supplementary Table 1**).

Flavonoids were only observed in measurable amounts for Am90, ranging from 1.32 – 3.34 µg g^-1^ dry mass. Treatment 1 had a significantly higher accumulation of flavonoids compared to all other treatments, although these significant differences were not detected when looking at isolated light spectral parameters (**Figure 4F**; **Supplementary Table 1**). Total phenolics covers the widest range of secondary metabolites and measures total reducing capacity of free radicals by phenolics and similar compounds. All three accessions had significant variation in total phenolics across the treatments (**Figure 4G**). In Am90, these results were significantly affected by DLI alone, while DLI and light spectral differences all had significant effects on Am25 and Am98 (**Supplementary Table 1**). Total phenolics values were highest under Treatment 2 for Am25 (10.88 ± 0.12 mg g^-1^ dry mass), Treatment 1 for Am90 (11.94 ± 0.42 mg g^-1^ dry mass) and Treatment 3 for Am98 (9.03 ± 0.36 mg g^-1^ dry mass). Am25 and Am90 had significantly lower total phenolics under Treatment 3 (6.67 ± 1.78 and 6.50 ± 1.76 mg g^-1^ dry mass, respectively), while Am98’s lowest total phenolics value was observed under Treatment 4 (5.35 ± 1.36 mg g^-1^ dry mass). Total phenolics across the three accessions followed the same trend of higher values under Treatments 1 and 2.

## Discussion

This study investigated the effects of red to blue (R:B) light ratios on hydroponically grown *Amaranthus tricolor* baby leafy greens in a growth chamber. Parameters under investigation included yield-related traits, photosynthetic pigments, and secondary metabolite accumulation including betacyanin and betaxanthin pigments, phenolic acids, and flavonoids. The four R:B ratio treatments included 1:1.6, 1:1.1, 1.5:1, and 1.9:1. The green waveband and DLI values were within 1 standard deviation for treatments 1-3, while treatment 4 had significantly lower green light and a significantly higher daily light integral (DLI) (**Table 1**; **Figure 1**). The results of this study confirmed that varying light spectral ratios have a significant impact on the accumulation of primary and secondary metabolites that control growth and development of certain genotypes of *Amaranthus tricolor* although these treatments did not significantly affect the overall yield of the plants.

Overall, plant yield was not significantly affected by the different R:B ratios applied. Across the yield-related traits evaluated in these three accessions studied, shoot height was significantly higher under Treatments 1 and 2 compared to Treatments 3 and 4 for Am25 (**Figure 2A**). This finding, however, is inconsistent with studies on other plant species, which suggest increased stem elongation correlates with higher red-light levels (e.g., Rehman et al., 2024; Wang et al., 2025). When examining the isolated light spectrum and DLI effects for shoot height in Am25, there were no significant differences observed, suggesting that higher blue light did not contribute to stem elongation, but some other unaccounted variable did (**Supplementary Table 1**). Overall, a significant difference was only observed for one of the three accessions with respect to shoot height. Therefore, a general conclusion cannot be made regarding the impact of R:B ratios on stem elongation in these three amaranth accessions. While Am98 did not have a significant overall treatment effect for leaf area, by isolating the effects of R:B, R:(G+B) ratios and DLI, there were significant differences observed for these individual parameters (**Supplementary Table 1**). Except for the leaf area of Am98, light spectrum or DLI did not appear to have an effect on measured yield-related traits, which might suggest that these ratios of R:B light were not varied enough to trigger significant responses in yield (**Figures 2B–D**).

Primary and secondary photosynthetic pigment compounds, chlorophyll a and b and carotenoids, were evaluated in this study as nutritional compounds. Across the four R:B ratio treatments, each of these compounds differed significantly within the three accessions (**Figure 3**; **Supplementary Table 1**). Chlorophyll a, the primary photosynthetic pigment absorbing light at 430 nm and 662 nm, was highest under Treatment 3 across all three accessions (**Figure 3A**). This is consistent with results reported in other studies that concluded that red light enhances chlorophyll content and photosynthetic rate in other plant species (e.g., Wang et al., 2025). Similar results were found for the accessory pigments chlorophyll b and carotenoids, which were all significantly higher under Treatment 3 (**Figures 3A, C**). Importantly, Treatment 4, with the highest R:B light ratio had lower levels of photosynthetic pigments, suggesting the importance of both red and blue light in the bioaccumulation of these compounds, rather than red light only. This emphasizes the importance of both red and blue light for photosynthetic pigment accumulation, though suggesting a greater importance for red light compared to blue light.

Phenolic acids, flavonoids, and betalain pigment compounds are secondary metabolites in *Amaranthus* spp. with important applications for human health. Betalain pigments include betacyanins (red/violet pigments) and betaxanthins (yellow/orange pigments) found in the *Caryophyllales* plant order, commonly found in food crops such as amaranth, beet root, and dragon fruit. These pigment compounds have been studied for their human health promoting and colorant properties and therefore understanding how to increase pigment production is important for amaranth. Am25 and Am98 produced red-colored leaves, while Am90 had green leaves with white stems, so the presence of both classes of betalain pigments was much greater and more varied in Am25 and Am98 (**Figures 4A, B**). These pigments were only significantly different in Am98, a completely red accession of amaranth. These pigments were most abundant under Treatments 1 and 3. Very few studies have looked at the impact of the PAR spectrum on betalain pigments, and if they did, they reported varying results, some found increased production under blue and UV lights (e.g., Kishima et al., 1995), while others reported an increase under more red light (e.g., Oh et al., 2022). The results from this study suggest that for accessions that produce higher amounts of betalain pigment compounds, the PAR spectrum does have an impact on biosynthesis, but further studies are needed to elucidate the specific effects.

Caffeoylglucaric acids, feruloylglucaric acids, coumaroylglucaric acids, caffeoylisocitric acids, and feruloylisocitric acids were the phenolic acid derivatives present in three *A. tricolor* cultivars, as were first identified by Strack et al. (1987), and further elucidated in recent years (Schröter et al., 2018; Engelhardt et al., 2023). These compounds were identified and quantified along with flavonoid quercetin and kaempferol derivatives in the three accessions evaluated in this study, and their biosynthesis was evaluated across the four different R:B ratio treatments (**Figures 4C–E**; **Supplementary Figure 2**). The phenolic compound quantities were summarized based on their bioactive precursor compounds, reported as caffeic acids, ferulic acids, and coumaric acids. Out of the three amaranth accessions evaluated, Am90 showed the most consistent significant differences across treatments for caffeic acids, ferulic acids and coumaric acids, but Am98 had more significant differences affected by light spectrum and DLI (**Figures 4C–E**; **Supplementary Table 1**). Although there was a consistent upward trend in phenolic acid accumulation in Treatments 1 and 2 compared to Treatments 3 and 4, these results may not have been attributed to differences in R:B ratio, but may have been impacted by other variables not accounted for. Flavonoid compounds were only present in measurable quantities in Am90, and were significantly higher under Treatment 1 with the highest amount of blue light (**Figure 4F**). Total phenolics encompassed the reducing capacity by phenolic acids and similar compounds within each amaranth accession, with a similar trend of higher amounts under treatments 1 and 2. DLI was the only significant variable for total phenolic differences in Am90, while light spectrum and DLI were all significant parameters for Am25. The higher levels of total phenolics under Treatment 3 for Am98 and Treatment 2 for Am25 could have been skewed by the abundance of betacyanin pigments, which were highest under Treatment 3 in both accessions (**Figures 4A, B, G**). Overall, accumulation of these nutritional secondary metabolites seem to be genotype-dependent and can be impacted by the PAR spectrum, with phenolic acids and flavonoids increasing in certain amaranth accessions that are subjected to higher levels of blue light.

## Conclusion

Overall, our study demonstrated genotype-dependent effects of light spectral ratios on *Amaranthus tricolor* baby greens grown in a controlled hydroponic environment. In this study, yield was not significantly impacted by changes in R:B ratios, while photosynthetic pigment and secondary metabolite concentrations demonstrated accession-dependent effects. This study was limited by the narrow change in R:B ratios applied across four treatments. The LED fixtures were only adjustable within the PAR range, so ultraviolet and far-red light effects could not be studied. A larger range of R:B ratios could increase the significance observed across treatments and the addition of UV light would have likely increased the secondary metabolite concentrations, which has been observed in other plants (Thoma et al., 2020). Our study suggests that the light spectrum has a significant impact on the accumulation of primary and secondary metabolites in the three amaranth accessions studied, with a negligible impact on yield characteristics. This is an important result when considering maximizing the nutritional content of leafy green amaranth without compromising the overall harvestable yield.

## Data Availability

The original contributions presented in the study are included in the article/**Supplementary Material**. Further inquiries can be directed to the corresponding author.
