# Supplementary material for: Impact of red-to-blue LED light ratios on the yield and phytochemistry of leafy green amaranths
Source: Front Plant Sci. 2026 Oct 30;17:1957692. doi: 10.3389/fpls.2026.1957692 (PMC13759789; doi:10.3389/fpls.2026.1957692)
Supplement: Supplementary file 1 [file DataSheet1.docx]

***Supplementary Tables and Figures***

**Supplementary Figure 1.** Average (n = 12) light spectra measured directly below each of the four LED fixtures at the top of the plant canopy. A. Week 1 and B. Week 2.

**Supplementary Figure 2.** Representative LCMS chromatograms of phenolic acids and flavonoids in three accessions (Am25, Am90, and Am98) of *Amaranthus tricolor*. The higher the peaks, the higher the content of the various compounds.

**Supplementary Table 1.** Comparative mixed model ANOVA P-values of yield and phytochemical traits of *Amaranthus tricolor* accessions. Model A analyzes overall treatment x accession effect. Model B analyzes R:B x R:G:B x DLI effect on significant differences.

| Trait | Model |  | Am25 | Am90 | Am98 |
| --- | --- | --- | --- | --- | --- |
| Shoot Height (cm) | A | Treatment | * |  |  |
|  |  | Accession | *** | *** | *** |
|  |  | Treatment x Accession |  |  |  |
|  | B | R:B |  |  |  |
|  |  | R:G:B |  |  |  |
|  |  | DLI |  |  |  |
| Fresh Mass (g) | A | Treatment |  |  |  |
|  |  | Accession |  |  |  |
|  |  | Treatment x Accession |  |  |  |
|  | B | R:B |  |  |  |
|  |  | R:G:B |  |  |  |
|  |  | DLI |  |  |  |
| Number of Mature Leaves | A | Treatment |  |  |  |
|  |  | Accession | *** | *** | *** |
|  |  | Treatment x Accession |  |  |  |
|  | B | R:B |  |  |  |
|  |  | R:G:B |  |  |  |
|  |  | DLI |  |  |  |
| Leaf Area (cm2) | A | Treatment |  |  |  |
|  |  | Accession | * | * | * |
|  |  | Treatment x Accession |  |  |  |
|  | B | R:B |  |  | * |
|  |  | R:G:B |  |  | * |
|  |  | DLI |  |  | * |
| Total Carotenoids | A | Treatment | *** | *** | *** |
|  |  | Accession | *** | *** | *** |
|  |  | Treatment x Accession | * | * | * |
|  | B | R:B |  | ** | ** |
|  |  | R:G:B |  | ** | ** |
|  |  | DLI |  | ** | ** |
| Chlorophyll A | A | Treatment | *** | *** | *** |
|  |  | Accession | *** | *** | *** |
|  |  | Treatment x Accession | * | * | * |
|  | B | R:B |  | ** | *** |
|  |  | R:G:B |  | ** | *** |
|  |  | DLI |  | ** | *** |
| Chlorophyll B | A | Treatment | *** | *** |  |
|  |  | Accession | *** | *** | *** |
|  |  | Treatment x Accession |  |  |  |
|  | B | R:B |  | *** |  |
|  |  | R:G:B |  | *** |  |
|  |  | DLI |  | *** |  |
| Total Chlorophyll | A | Treatment | *** | *** | *** |
|  |  | Accession | *** | *** | *** |
|  |  | Treatment x Accession |  |  |  |
|  | B | R:B |  | ** | ** |
|  |  | R:G:B |  | ** | ** |
|  |  | DLI |  | ** | ** |
| Betacyanins | A | Treatment |  |  | * |
|  |  | Accession | *** | *** | *** |
|  |  | Treatment x Accession |  |  |  |
|  | B | R:B |  |  | * |
|  |  | R:G:B |  |  | * |
|  |  | DLI |  |  | * |
| Betaxanthins | A | Treatment |  |  | * |
|  |  | Accession | *** | *** | *** |
|  |  | Treatment x Accession |  |  |  |
|  | B | R:B |  |  | * |
|  |  | R:G:B |  |  | * |
|  |  | DLI |  |  | * |
| Caffeic Acid | A | Treatment | * | * |  |
|  |  | Accession | * | * | * |
|  |  | Treatment x Accession |  |  |  |
|  | B | R:B |  |  |  |
|  |  | R:G:B |  |  |  |
|  |  | DLI |  |  |  |
| Ferulic Acid | A | Treatment |  | *** | *** |
|  |  | Accession | *** | *** | *** |
|  |  | Treatment x Accession | *** | *** | *** |
|  | B | R:B |  |  | ** |
|  |  | R:G:B |  |  | ** |
|  |  | DLI |  | * | ** |
| Coumaric Acid | A | Treatment |  | ** | ** |
|  |  | Accession | *** | *** | *** |
|  |  | Treatment x Accession |  |  |  |
|  | B | R:B |  |  | ** |
|  |  | R:G:B |  |  | ** |
|  |  | DLI |  |  | ** |
| Total Flavonoids | A | Treatment |  | * |  |
|  |  | Accession | *** | *** | *** |
|  |  | Treatment x Accession |  |  |  |
|  | B | R:B |  |  |  |
|  |  | R:G:B |  |  |  |
|  |  | DLI |  |  |  |
| Total Phenolic Acids | A | Treatment | *** | *** | *** |
|  |  | Accession | ** | ** | ** |
|  |  | Treatment x Accession | ** | ** | ** |
|  | B | R:B | * |  |  |
|  |  | R:G:B | * |  |  |
|  |  | DLI | * | * |  |

* signifies P < 0.05, ** signifies P < 0.01, *** signifies P < 0.001
